# 

**Published:** 1852-07-01

**Authors:** 


IT is our intention to publish in the next Number a large body of miscellaneous matter, and analysis of several foreign journals and works.

